# Intensive home treatment for patients in acute psychiatric crisis situations: a multicentre randomized controlled trial

**DOI:** 10.1186/s12888-018-1632-z

**Published:** 2018-02-27

**Authors:** Jurgen Cornelis, Ansam Barakat, Jack Dekker, Tessy Schut, Sandra Berk, Hans Nusselder, Nikander Ruhl, Jeroen Zoeteman, Rien Van, Aartjan Beekman, Matthijs Blankers

**Affiliations:** 1 Department of Research, Arkin Mental Health Care, Klaprozenweg 111, 1033 NN Amsterdam, The Netherlands; 2 Department Clinical Psychology, VU University, Van der Boechorststraat 1, 1081 BT Amsterdam, The Netherlands; 3 Department of Emergency Psychiatry, Arkin Mental Health Care, Amsterdam, The Netherlands; 4 Department of Emergency Psychiatry, GGZ inGeest Mental Health Care, Amsterdam, The Netherlands; 5 GGZ inGeest and Department of Psychiatry, Amsterdam Public Health research institute, VU University Medical Center, A.J. Ernststraat 1187, 1081 HL Amsterdam, The Netherlands; 6 Academic Medical Center, Department of Psychiatry, University of Amsterdam, Amsterdam, The Netherlands; 7 Trimbos Institute – The Netherlands Institute of Mental Health and Addiction, Da Costakade 45, 3521 VS Utrecht, The Netherlands

**Keywords:** Crisis resolution, Community mental health, Psychiatric crisis, Cost-effectiveness, Admission days, Randomized controlled trial

## Abstract

**Background:**

Hospitalization is a common method to intensify care for patients experiencing a psychiatric crisis. A short-term, specialised, out-patient crisis intervention by a Crisis Resolution Team (CRT) in the Netherlands, called Intensive Home Treatment (IHT), is a viable intervention which may help reduce hospital admission days. However, research on the (cost-)effectiveness of alternatives to hospitalisation such as IHT are scarce. In the study presented in this protocol, IHT will be compared to care-as-usual (CAU) in a randomized controlled trial (RCT). CAU comprises low-intensity outpatient care and hospitalisation if necessary. In this RCT it is hypothesized that IHT will reduce inpatient days by 33% compared to CAU while safety and clinical outcomes will be non-inferior. Secondary hypotheses are that treatment satisfaction of patients and their relatives are expected to be higher in the IHT condition compared to CAU.

**Methods:**

A 2-centre, 2-arm Zelen double consent RCT will be employed. Participants will be recruited in the Amsterdam area, the Netherlands. Clinical assessments will be carried out at baseline and at 6, 26 and 52 weeks post treatment allocation. The primary outcome measure is the number of admission days. Secondary outcomes include psychological well-being, safety and patients’ and their relatives’ treatment satisfaction. Alongside this RCT an economic evaluation will be carried out to assess the cost-effectiveness and cost-utility of IHT compared to CAU.

**Discussion:**

RCTs on the effectiveness of crisis treatment in psychiatry are scarce and including patients in studies performed in acute psychiatric crisis care is a challenge due to the ethical and practical hurdles. The Zelen design may offer a feasible opportunity to carry out such an RCT. If our study finds that IHT is a safe and cost-effective alternative for CAU it may help support a further decrease of in-patient bed days and may foster the widespread implementation of IHT by mental health care organisations internationally.

**Trial registration:**

The trial is registered in the Netherlands Trial Register as # NTR-6151. Registered 23 November 2016.

## Background

For many years hospitalisation has been the indicated care modality for patients experiencing a psychiatric crisis. Alternatives are available [1, 2] such as treatment by outpatient Crisis Resolution Teams (CRTs) [3–6] which are named Intensive Home Treatment (IHT) teams in the Netherlands [7].

There are several arguments from professionals and patient movements to treat people in the community and not in the hospital. Hospital admission can be harmful and stigmatizing to patients while relationships between patients, their relatives and professionals are different and less dominated by inequalities of power when crises are managed in the patients’ own homes.

Social and environmental triggers can contribute to a crisis. By visiting the patient’s home, these triggers can better assessed and addressed. Learning coping skills can be more effective in the environment where these are needed and can help to prevent future crises. Professionals can better respond to the needs of patients and their relatives if they meet them in their own social environment [8, 9]. In addition to these arguments, economic and political pressure encourages community mental health care to develop further. Our working definition of a psychiatric crisis is a situation in which the severity of current acute clinical and social problems and associated risks indicate that admission to an acute psychiatric ward is necessary [10].

Treating patients during a psychiatric crisis may entail intensifying already existing care provided by community mental health teams as in (Functional) Assertive Community Treatment ((F)ACT) teams [11–13]. This care modality is most often prescribed for patients with a disabling Severe Mental Illnesses (SMI), who need intensified care for a long duration of time. But there are also patients experiencing a psychiatric crisis with a need for care of varying intensity. For those patients CRTs can provide an acute intensive specific short-term crisis treatment in their home setting [6]. With such an intervention, patients can stay in their own social environment, while psychiatric and social problems can be assessed and addressed by professionals in cooperation with family or friends of the patient.

### Current evidence base

In a recent Cochrane review on psychiatric crisis interventions [14] eight RCT studies have been included. In five of the eight studies, crisis care was part of the overall care package, while in two studies a telephone answering service comprised the crisis resolution intervention. Treatment of a psychiatric crisis by a specialized CRT team was to our knowledge only investigated in the RCT of Johnson et al. (2005) [15]. Johnson and colleagues compared CRT with CAU, consisting of hospitalisation or a community mental health approach. They found a significant difference in the number of patients hospitalised at 8 weeks (IHT: 36%, CAU: 69%, *p* < 0.0005) and 6 months (CRT: 47%, CAU: 75%, p < 0.0005) after crisis initiation. Eight weeks after crisis initiation, outpatient crisis care improved the mental and social well-being of patients more than CAU, as measured by the Health of the Nation Outcome Scale (HoNOS). However, this effect evaporated 6 months after crisis initiation [15].

Quasi-experimental research, often a comparison between an area with and without CRT, and pre-post CRT care evaluations found reduced hospitalization rates following CRT [5, 16, 17] and fewer days in the hospital [14, 17] when compared to care without a CRT service. Clinical and social outcomes were similar in CRT conditions compared with non-CRT care [18]. Based on pre-post intervention evaluations, Kilian et al. (2016) found that CRT was more effective than admission with respect to HoNOS scores (b = − 4.43; *p* = 0.02) and reductions of depressive symptoms measured by the Hamilton Depression scale (HAMD) (b = − 4.11; *p* = 0.004), but not with regard to the total score of psychotic symptoms measured by the Positive and Negative Syndrome Scale (PANNS) (b = − 4.51; *p* = 0.134). Outpatient crisis care was more acceptable and satisfactory to service users [17, 19], their families and other relatives and reduced the stigmatization inherent to hospitalisation [20] than inpatient care. Burden (such as disruption to daily routine, social life and susceptibility to physical illness) was also reduced for out-patients, their families and other relatives [14, 20].

A recent prospective cohort study performed in Germany included 118 patients with acute mental disorders and found early evidence for the cost-effectiveness of CRT teams compared to hospitalisation [21]. However, methodological limitations, such as different target populations in different studies and variations in study designs complicate the interpretation of the findings [5, 17, 19]. Given the limited evidence base, there is an urgent need for more effectiveness studies on CRT.

## Methods/design

### Research aims and hypotheses

The primary objective of this study is to test whether IHT reduces in the number of inpatient days per patient in the year following psychiatric crisis compared to CAU. Based on previous research [14, 15, 22, 23] and the naturalistic IHT project evaluation in Amsterdam [24] we hypothesize that IHT will lead to a 33% reduction in the number of hospitalisation days at 52 weeks post-randomisation, compared to CAU. This translates to an average reduction of 20 hospitalisation days: 40 days (SD = 40) for IHT and 60 days (SD = 55) for CAU.

### Secondary hypotheses

- *Safety.* With regard to safety it is expected that patients in the IHT condition will report the same amount or less symptoms of social malfunctioning and aggression than CAU patients [17].
- *Psychiatric symptoms*. With regard to psychiatric symptoms it is expected that participants in the IHT condition will report the same amount or less symptoms than CAU patients [15].
- *Treatment satisfaction.* Higher treatment satisfaction treatment of the participants and their relatives [20, 25] in the IHT is expected compared to CAU.

Alongside this RCT, an economic evaluation will be performed in order to assess the cost-effectiveness and cost-utility of IHT compared to CAU.

### Design

The trial in this protocol is designed as a 2-centre, 2-arm Zelen double consent open label RCT, to test the efficacy and cost-effectiveness of IHT against CAU. This design is chosen after careful consideration and fruitful discussions between the researchers and the IHT professionals and has been approved by the Medical Ethics Committee of VU University Amsterdam (MEtC VUmc) as #NL55432.029.16.

During the first contact with professionals following the start of a psychiatric crisis patients are randomly allocated to one of the two treatment modalities, IHT or CAU. This allocation takes place in a 2 (IHT): 1 (CAU) ratio (for reasons of staff and facility capacity). Consent to participation in the trial and to treatment allocation will be sought as soon as the psychiatric condition of the patients is stabilized, ultimately 3 weeks after intake.

According to the Zelen double consent design all participants will be fully informed about the study during the first meeting with a member of the research group and before consent is sought [26]. This implies that they get information about treatment allocation based on pre-randomisation, study procedures, treatment options, and the possibility to cross-over to the other treatment condition, before they are asked to sign the informed consent form. Only patients who provide written informed consent are included in the study. Participants who cross over between the two conditions will be followed up and analysed as members of the trial arm to which they were originally allocated according to the intention to treat principle [27].

### Study procedure

#### Recruitment

Arkin and GGZ inGeest are the two largest mental health care organisations in Amsterdam, the Netherlands and provide in- and outpatient psychiatric care in Amsterdam for children and adults of all ages. The Psychiatric Emergency Service Amsterdam (PES) is a cooperation between these two organisations. Referrers to PES include general practitioners, other mental health professionals and the police. A professional of PES performs psychiatric assessments, prepares a treatment plan with the patient in cooperation with his family and other professionals, and provides care as described in the treatment plan. PES is available 24 h a day, 7 days a week. The IHT teams are part of PES.

In this study, participants are recruited via IHT teams and the in-patient facilities of Arkin and GGZ inGeest between 8.30 AM and 10.00 PM. During the night, between 10:00 PM and 8:30 AM, PES Amsterdam is functioning as a gatekeeper for Arkin and GGZ inGeest. During night hours patients assessed by this service will receive CAU, i.e. voluntary or involuntary psychiatric admission or community-based care, with support from PES if necessary. The next day, these patients will be screened to check if they meet the in/exclusion criteria for this study. If participants have been admitted to a psychiatric ward during the weekend, they will be screened for study participation the next working day.

#### Participants

All patients are recruited from mental health care organisation Arkin and InGeest in Amsterdam which provide high intensive psychiatric care, IHT treatment or admission to a psychiatric ward. For this study the following in- and exclusion criteria apply:

#### Inclusion criteria

- A psychiatrist must state that admission to a clinical crisis care unit is indicated, if necessary on a compulsory basis. This decision is based on clinical outcomes, judgments of other professionals and information from informal caregivers
- Patient is diagnosed with at least one axis I or II disorder
- The patient is a resident of Amsterdam, Diemen or Driemond, the Netherlands.
- Age between 18 and 65 years old
- Ability to read and understand the Dutch language
- Written informed consent has been provided by patient

#### Exclusion criteria

- Patient is homeless
- Primary diagnosis of the patient is substance use disorder for which referral to a specialized treatment unit is indicated
- Patient is currently receiving (F)ACT care
- Patient has previously received IHT treatment

#### Screening and pre-randomisation

Patients will be screened and pre-randomised by members of the IHT teams or by the patient placement desks of the treatment organisations. For the members of these teams, an online pre-randomisation tool to check whether the in- and exclusion criteria are met has been developed. Patients who do not meet the in- and exclusion criteria will not be included in the study and receive any treatment deemed necessary. Patients who meet the in- and exclusion criteria will be pre-randomised to IHT or CAU.

Pre-randomisation is performed using a seeded pseudo random number generator (Mersenne twister). The seed is based on patient characteristics, to ensure that a given patient is allocated to the same trial arm in case the randomisation procedure would be run more than once. This is necessary because it sometimes happens that in the midst of a psychiatric crisis two or more health care workers may fill out the screening and allocation tool for the same person. The outcome of the pre-randomisation is not only known by the one who is pre-randomising but the outcome is also sent to members of the research team.

#### Consent and mental competence

After allocation, researchers will get in contact with the IHT team or ward treating the patient and ask a doctor or nurse about the patient’s mental competence to give informed consent for the study. At admission, and within 24 h after screening and pre-randomisation, the mental competence of the patient will be assessed by a clinician using a tool designed by the Dutch federation of medical doctors (Koninklijke Nederlandsche Maatschappij tot bevordering der Geneeskunst), the KNMG tool [28]. If there is any doubt about the patients’ mental competence during this first assessment, the patient will be assessed again by a clinician within two weeks. For this assessment the clinician may use the Dutch translation of the MacArthur Competence Assessment Tool-Clinical Research (MacCAT-CR) ([29], Hein I. MacCAT-CR: manual and sample interview (translation). [MacCAT-CR Handleiding en voorbeeldinterview (vertaling) (in Dutch)]. 2017. Not published).

The research team will contact the patient after the clinician evaluates him/her as mentally competent to give informed consent and then the informed consent procedure will take place. If a patient is still not deemed mentally competent to give informed consent 5 weeks after the start of the crisis treatment, (s)he will not be included in the study.

All patients who meet the inclusion criteria, none of the exclusion criteria, and who are mentally competent to give an informed consent will be approached by the research team and receive information about the study. The patient and his relatives will receive an information folder describing the CAU and IHT interventions and study procedures. An appointment will be made to inform the patient about treatment allocation, answer any questions the patient may have regarding the study and to collect the written informed consent if possible. Consent will be sought regarding (1) the treatment condition they are allocated to, (2) the baseline and follow-up measurements, (3) to inform their relatives, (4) informing their general practitioners, other mental health professionals, (5) informing and asking their pharmacist about drug use, (6) to request and use their data from Statistics Netherlands (Centraal Bureau voor de Statistiek (CBS)). A patient can also participate in this study by giving informed consent to using existing data sources such as their Electronic Health Record (EHR) and CBS data only – EHR data is sufficient to test our primary hypothesis (number of hospitalisation days).

If the patient hesitates about providing informed consent, (s)he will have 5 more days to decide to participate. Only after providing written informed consent, baseline measurements are administrated. If a patient decides not to provide informed consent, (s)he is not included in the study (and can receive any treatment deemed necessary).

#### Blinding

After giving this consideration, we think that is it not practically feasible to effectively blind the patient, therapist or researcher to the allocated treatment condition. Patients will be fully informed about the treatment options before providing informed consent. Researchers will have contact with the therapist about mental competency for informed consent, and hence will know whether the therapist is working on a ward or for an IHT-team. Information about whether the patient resides on the psychiatric ward or at home gives an indication of the allocated treatment. Therefore we have designed this RCT as an open label study.

### Interventions

#### Intensive home treatment

IHT is a treatment modality that addresses some of the imperfections of inpatient care by providing care in the patients’ home setting in the least restrictive environment; ‘normal’ life is disrupted as little as possible. In this way collaboration with and utilization of the patient’s social system can be maximised. During the first contact, patient, professionals and the most involved members of the patient’s social system discuss the problems or goals to be addressed during treatment. Collectively the priority of aims to achieve will be determined. In addition, the ways in which to achieve the aims, who takes responsibility for which part of the treatment plan and with whom the progress will be evaluated after 3 and 6 weeks is collectively decided. IHT professionals have the capacity to visit the patient at home, up to three times per day. The patient (and his/her relatives) can reach the IHT professionals 14 h per day, seven days a week (from 8.30 AM till 10:00 PM), while PES can be reached outside these hours if necessary. The IHT team starts as soon as possible after treatment allocation and remains involved until the crisis is resolved, for an average duration of 6 weeks. After IHT, the patient is referred to appropriate follow-up treatment if necessary.

The IHT team is a multidisciplinary team consisting of a psychiatrist, a psychiatry resident, a psychologist and (social) psychiatric nurses. All members of the team received additional education in family and relational treatment. Professionals of the IHT can provide:

- Assessment: psychiatric diagnostic, risk taxation
- Psycho-education to the patient and his relatives
- Support in structuring patient’s daily life, and in solving social and financial problems
- Supportive and cognitive behavioural interventions
- Pharmacotherapy
- Support and empowerment to patient’s informal care system so they will be able to sustain care
- Support a more gradual transition between in-patient care and low intensity out-patient care if the patient is admitted to a psychiatric ward
- Referral, if necessary, to specific treatment settings

#### Care as usual

Standard crisis care often includes admission or low intensity (i.e. less than 3 times a week) outpatient treatment. If admitted, the patient’s environment is generally highly structured at the outset (a closed ward, for example), and becomes less structured as the patient’s condition improves (open ward). This may be followed by day treatment and follow-up treatment in an outpatient department [30, 31]. Patients in the CAU condition typically receive a combination of inpatient (first phase) and low-intensity outpatient (second phase) treatment. The length of the different phases depends mostly of the severity of the symptoms, presence of danger, social factors like housing and availability of support by family and friends. Professionals at a psychiatric ward are psychiatrists, medical doctors, psychologists, nurses and other mental health professionals. No treatments are withheld from patients in CAU except IHT.

The contents of treatment can be similar as provided by the IHT professionals but in a different setting. Benefits for the patient of being admitted can be:

- The feeling of being in a safe environment because professionals are near
- Not being dependent on help from family or friends
- Being liberated from role obligations present in the home setting

### Measurements

Measurements will be conducted at the start of the treatment (T0) and at 6, 26 and 52 weeks follow-up (T1, T2, T3; see Table 1). All self-report questionnaires, potential effect moderators and demographic variables will be collected using several assessment methods. Once written informed consent has been provided, the research team will conduct the data collection of the participating patient through structured interviews and by collecting specific data from the EHR system in both mental health care organisations. The data collection of the relative and professional involved in the treatment will be conducted using a web-based platform. From the pilot experience it is known that the baseline interview will take about two hours and that the follow-up interviews take about one and a half hour to complete. To compensate patients for their time and efforts, patients receive a remuneration of €10 for the completed baseline questionnaire. For the follow-up questionnaire the patient will receive a remuneration of €15 (6 weeks), €20 (26 weeks) and €25 (12 months).

**Table 1:** Measurement instruments

					Timetable (weeks)			
Categories	Subcategories	Instruments	About	By	Base-line	6	26	52
Demographics	Diversity of questions	–	Pat & Re	All	x			
Safety	Harm, violence, and suicide	SDAS (short)	Pat	Prof.	x	x		
Psychiatric symptoms	Diagnostic Classification	DSM-IV-TR /V	Pat	Prof.	x			
	Psychosocial functioning	HoNOS	Pat	Prof.	x	x		
	Symptoms	BSI (short)	Pat	Res	x	x		x
	Symptoms	BPRS	Pat	Res	x	x	x	x
Treatment satisfaction	Patients’ satisfaction	ZUF-8	Pat	Res		x		x
	Patients’ treatment satisfaction	TPQ	Pat	Res		x		
	Relatives’ satisfaction	CIS	Re	Re		x		x
	Professionals’ satisfaction	Self-developed	Prof.	Prof.		x		
Social support & Network extent	Patients’ perspective	Personal recovery	Pat	Res	x	x	x	x
	Relatives’ perspective	BES	Re	Re	x	x		x
Self-efficacy		MHCS	Pat	Res	x	x	x	x
Quality of life		EQ-5D-5L	Pat	Res	x	x	x	x
		SF-12	Pat	Res	x		x	x
		MANSA	Pat	Res	x			x
Substance use	Alcohol	AUDIT	Pat	Res	x		x	x
	substance abuse	MATE-I	Pat	Res	x		x	x
Cost-effectiveness	direct medical contacts	TiC-P	Pat	Res	x		x	x
	Delivered informal care and support by relative	iVICQ (short)	Re	Re	x	x		x
	Absenteeism from work	PRODISQ (short)	Pat	Res.	x		x	x
Supplementary	Symptoms	S&P	Pat	Prof.	x			

Pat = participant/patient; Re = relative; Prof. = professional; Res = researcher; Self = self report; Int = Interview

AUDIT = Alcohol Use Disorder Identification Test (AUDIT); BES = Betrokkenen Evaluatie Schaal [Stakeholders Evaluation Scale]; BPRS = Brief Psychiatric Rating Scale; BSI = Brief Symptom Inventory; CIS = Contact, Information and Support; CSQ = Client Satisfaction Questionnaire; DSM = Diagnostic and Statistical Manual of Mental Disease; EQ-5D-5L = European Quality of Life, 5 Dimensional, 5 level; HoNOS = Health of the Nation Outcome Scale; iVICQ = iMTA Valuation of Informal Care Questionnaire; MANSA = Manchester Short Assessment of Quality of Live; MATE = Measurements in the Addictions for Triage and Evaluation; MHCS = Mental Health Confidence Scale; PRODISQ = Productivity and Disease Questionnaire; SDAS = Social Dysfunction and Aggression Scale; SF-12 = Short Form health survey; S&P = Staging and Profiling; TPQ = Treatment Perception Questionnaire; TiC-P = Trimbos Institute and Institute of Medical Technology Questionnaire for Costs Associated with psychiatric Illness; ZUF-8 = Zufriedenheit-8 (Satisfaction scale)

The informatics team in both mental health care organisations will assist in data collection from the EHR system. The research team provides a list of participants’ surnames, date of birth, a unique project number for each participant and a standardised schedule of the information required for each participant. The required data will be specified with the time period for which data is needed. By providing the data only by research number data risks from transferring identifiable patient data will be minimized.

### Demographic variables

At baseline, participants complete questions concerning basic demographic details; those questions include age, gender, country of origin, parents or relative’s country of origin, domestic situation, education level, occupational status and income. Drug history will be requested from the pharmacy (with participant’s consent), while the use of mental health care in the year before allocation to this study will be collected from the EHR system. The use of other health care will be collected from the CBS data (with participant’s consent) and the TiC-P instrument.

### Primary outcome measure

The number of hospitalisation days in the 52 weeks after randomisation is the primary outcome measure. This includes the duration of the initial admission at baseline, and any subsequent psychiatric admission during the 12 months follow-up period of the study. Data will be collected from the EHR system of both mental health care organisations.

### Secondary outcome measures

1. Safety:

Social Dysfunction & Aggression Scale (SDAS); this 4-item questionnaire assesses harm, including self-inflicted, violence, and suicide attempts [32].

2) Psychiatric symptoms:
  - DSM-IV or V classification [33, 34].
  - Health of the National Outcomes Scales (HoNOS); 15-item scale measure of the health and social functioning of people with severe mental illness [35–37].
  - Brief Symptom Inventory (BSI) ; 53-item self-reported 5 point (0–5) Likert-type scale measuring the extent to which individuals have been disturbed by certain mental health symptoms [38].
  - Brief Psychiatric Rating Scale (BPRS); 24-item interview based scale to measure psychiatric symptoms [39, 40].
3) Treatment satisfaction:
  - The Client Satisfaction Questionnaire (CSQ): Zufriedenheit 8 items (ZUF-8) [41, 42], an open access variant of the CSQ-8 [43]) and the Treatment Perceptions Questionnaire (TPQ) [42, 44, 45].
  - Family Contact, Information and Support (C.I.S.) Questionnaire. Relatives’ satisfaction about patients’ treatment and the contact with the professionals is assessed using the questionnaire [46].
  - The professional will be asked to give on a 10-point scale an appreciation of the treatment. This self-developed questionnaire assesses overall treatment satisfaction, treatment compliance and treatment plan and healthcare process.

### Other outcomes of interest

1. Social support and network:
  - Personal recovery list and the Network and Support questionnaire. Those two self-developed questionnaires assess the patients’ perspective of social support and network.
  - Relatives Evaluation scale (Betrokkenen Evaluatie Schaal (BES) in Dutch): relatives’ perspective support will be assessed using a short version of this questionnaire, existing of 22 items [47].
2. Self-Efficacy:
  The Mental Health Confidence Scale (MHCS); 16-items questionnaire developed to measure self-efficacy in individuals diagnosed with mental disorders [48].
3. Quality of life:
  - Short Form health survey (SF-12); a set of 12 items measure the quality-of-life [49].
  - European Quality of Life-5 Dimensions (EQ-5D-5L); a 6-item self-report about quality of health [50, 51].
  - Manchester Short Assessment of Quality of Life (MANSA); a 16-item self-report [52].
4. Alcohol and substance use:
  - Alcohol Use Disorder Identification Test (AUDIT); an 10-item self-report about the use of alcohol [53]
  - Measurements in the Addictions for Triage and Evaluation (MATE-Interview) [54].
5. Cost-effectiveness:
  - EuroQol (EQ-5D-5L), 5-point scale used to measure quality of life [55].
  - Trimbos Institute and Institute of Medical Technology Questionnaire for Costs Associated with Psychiatric Illness (TiC-P) [56] and PROductivity and DISease Questionnaire (PRODISQ), short version [57]. Those questionnaires volume health care consumption and productivity loss.
  - Institute for Medical Technology Assessment (iMTA) Valuation of Informal Care Questionnaire (iVICQ); 11-item questionnaire volumes delivered informal care and support by relatives [58].
6. Staging and profiling:
  - The Staging and Profiling scale (S&P) is a self-developed measure assessed by health care professionals for the duration and the severity of the illness.

### Handling and storage of data and documents

Once the participating patient provides written informed consent, a unique project number will be allocated to each participant. The key of these project numbers will only be available to the principal investigators, the relevant data managers and research assistants. All (paper and digital) questionnaires and data will be stored and handled using this project number (de-identified). Study outcomes will be reported anonymously. Storage of data will be supervised by the principal investigator and complies with the Dutch Personal Data Protection Act.

### Data analysis

#### Sample size calculation

We expect a 33% reduction in hospitalisation days at 52 weeks post-treatment allocation in IHT compared to CAU. This translates into a reduction of 20 hospitalisation days: 40 days (SD = 40) for IHT compared to 60 days (SD = 55) for CAU. Given the fact that data on the number of hospitalisation days will be collected (with permission) from the EHR of the participating treatment centres, a low non-response rate is expected: we expect that up to 5% of the participants will actively withdraw during follow-up based on previous studies performed by our research group [24]. Based on these figures, a sample size calculation using R version 3.2.2 software and the power calculation package ‘pwr’ was performed for the inter-group difference in hospitalisation days at 52 weeks after treatment allocation. Alpha was set at 0.05 (2-sided), power was set at 0.80. In addition, to ensure that a loss of statistical power does not occur due to cross-over of randomly allocated participants, 5% will be added to the calculated sample size [59]. A 2:1 allocation ratio will be applied because for reasons of staff and facility capacity. Taking into account these parameters, we need to include a total of *n* = 230 patients. 153 of them need to be allocated to and provide informed consent for IHT, 77 need to be allocated to and provide informed consent for CAU.

#### Cost-effectiveness calculations

The economic evaluation will be conducted as a cost-utility analysis using quality adjusted life years (EQ-5D/QALYs) as a generic measure of health gains. We will use the EQ-5D tariffs from both the UK [60] and the Netherlands [61]. Health utilities based on the SF-12 will be calculated using the Brazier’s algorithm [62]. The primary endpoint will be 12 months post allocation for the cost-utility analysis. The cost-utility analysis will be conducted and reported in agreement with the Consolidated Health Economic Evaluation Reporting Standards (CHEERS). The trial’s follow-up measurements do not exceed the time horizon of one year and therefore neither costs nor effects will be discounted. Sensitivity analyses will be directed at uncertainty in the main cost drivers. We will consider five types of costs: (1) the costs of offering the intervention, (2) costs stemming from health care uptake (e.g. hospitalisation) including the costs of medication, (3) patients’ out-of- pocket costs, (4) costs stemming from productivity losses due to absenteeism and reduced efficiency while at work (if the patient holds a job) and 5) costs of informal care network. We will perform the cost-utility analysis from at least two perspectives: (1) Societal perspective, (2) healthcare provider perspective. Volumes of direct medical contacts will be collected using the TIC-P and PRODISQ-short version, using the EHR of the participating institutions, and using external data sources such as from CBS if possible. These different but overlapping sources will be used in order to assess the reliability of the data sources. Delivered informal care and support by relatives will be measured using the iVICQ-short version. Unit costs for delivered care will be obtained from most recent costing manuals for the societal perspective, and unit costs for the healthcare provider perspective will be obtained from the Dutch Health Care Authority (NZa) averaged costs. Data presented will include incremental cost-effectiveness ratio’s (incl. Gains in QALY), and cost effectiveness acceptability curves. Uncertainty around the reported parameters will be assessed using non-parametric bootstrapping.

Analyses will be performed according to the intention-to-treat principle, with missing data addressed using multiple imputations [63]. Incremental cost-effectiveness ratio (ICER) will be calculated as follows: ICER = (CI1-CI2)/(EI1-EI2), where Care are costs, E effects, and subscripts I1 and I2 refer to the two interventions (IHT and CAU). Confidence intervals around the ICER will be calculated using a non-parametric bootstrap approach: 1000 non-parametric bootstrapped samples will be extracted from each of the original datasets. For each of these bootstrapped samples, the incremental costs, incremental effects, and the ICER will be calculated. The resulting 1000 ICERs per dataset will be used for further calculations and will be plotted on a cost-effectiveness plane. Based on the distribution of the ICERs over the cost-effectiveness plane, cost-effectiveness acceptability curves (CEACs) will be drawn. The CEACs show the probability that IHT is more cost-effective than CAU, as a function of the willingness to pay (WTP) for one additional unit of effect (e.g. for one QALY gained). One-way sensitivity analyses directed at uncertainty in the main cost drivers will be performed to gauge the robustness of our findings across a range of likely values of those parameters.

### Statistical analysis

The primary analyses will be conducted according to the intention to treat principle. A per protocol/treatment completer analysis will also be conducted. Generalized Linear Mixed Models (GLMM) will be used to model the primary outcome measure (hospitalisation days after 12 months, (over-dispersed) Poisson distribution) and secondary outcome measures (distributions as appropriate). Psychiatrist and organisation (Arkin or GGZ inGeest) will be included as random factors in the model. Missing data will be addressed using multiple imputation. All analyses will be performed using Statistical Package for the Social Science (SPSS) version 23 or higher, and using R (and relevant packages) version 3.2.2 or higher.

### Monitoring, quality assurance and approvals

The study will be conducted in accordance to Good Clinical Practice guidelines and the Helsinki declaration. It is possible that the research project will be audited. During an audit an independent researcher will evaluate whether the study is being or has been performed in compliance with the quality standards of the Good Clinical Practise guidelines. Ethical approval has been obtained from the Medical Ethical Committee (METc) of the Vrije Universiteit medical centre Amsterdam (METc VUmc). This committee have approved the protocol and all amendments. If applicable, future amendments will be sent to the METc VUmc. In addition, the research team will submit a summary of the progress of the manuscript to the METc VUmc and the funder Stichting tot Steun VCVGZ once a year. Information will be provided on the date of inclusion of the first subject, numbers of subjects included and numbers of subjects that have completed the trial, serious adverse events/ serious adverse reactions, other problems, and amendments.

## Discussion

The study presented in this protocol paper is among the first RCTs worldwide to test the (cost-) effectiveness of 6 weeks of IHT compared to CAU (in- and out-patient crisis care without IHT teams). Primary outcome is the number of inpatient days compared to CAU, 12 months after randomisation. Secondary outcomes include safety of the patient, psychiatric symptoms and treatment satisfaction of the patient, his relatives and the professional.

Due to convictions of professionals and patients, “patients heal better in their own environment”, partially supported by previous research (e.g. [15]), economic and political pressure, IHT has been developed and may be broadly implemented without strong research evidence. The results of this study may ultimately help provide an evidence base to support or challenge the further implementation of IHT.

The design of this study must be evaluated in the light of the following strengths and challenges. Major strengths of this study are the follow-up period of 12 months and the relatively large sample size of 230 psychiatric crisis care patients. A major challenge is to perform an RCT in the acute mental health care setting. This challenge has led us to adopt the Zelen double consent design. At first we have considered the preferred RCT design: first obtaining informed consent followed by randomisation. But after discussions with the IHT professionals this design seemed impossible to implement in this study for a number of reasons:

- Patients in this study are in the midst of a psychiatric crisis. Patients have severe psychiatric symptoms, sometimes accompanied with severe danger for themselves or their network. They often experience a very emotional phase in their lives and are often surrounded by emotionally exhausted relatives. In this situation it is in our opinion unethical to ask for informed consent if this would delay a patient’s treatment.
- If treatment allocation by randomisation would take place after informed consent, patients and relatives are confronted with the fact that allocation depends on the outcome of a chance event. This may negatively influence their confidence in the selected therapeutic approach – even though there is no indication that one is superior to the other.
- Some patients lack insight in their illness in the most acute phase of the psychiatric crisis and therefore refuse treatment and do not want to cooperate with treatment and a study that is conducted to measure its effect.
- One of the powerful elements of the IHT intervention, i.e. the immediate initiation of patient’s and his relatives’ motivation for home treatment, would be weakened if consent would have to be sought prior to the start of treatment.

The research group therefore concluded that the pre-randomised Zelen double design would be a better and more feasible study design than the traditional RCT with consent before randomisation. Under the Zelen design, patients are randomised before consent to participate has been sought; so called pre-randomisation. In the Zelen double consent design all patients of both groups, the experimental and control group, will be fully informed about all aspects of the trial, including both treatment conditions, as soon as possible. Treatment starts immediately as the patient is presented to the professionals, without potential delays due to research consent procedures.

In addition to this allocation challenge that has led us to adapt the Zelen design, a number of practical challenges may arise while performing this study. For example, professionals may be biased towards one of the treatment conditions – which may impact the patient and his/her support for the provided treatment modality. In order to prevent this from impacting our study, we have frequent meetings with care professionals in order to answer their questions, resolve any worries, and to keep them positively involved in the study. Another challenge is the timing of the assessment of patients’ mental competence to provide informed consent. In case of doubt regarding this mental competence by the treatment professionals or the researchers, we use a structured and validated instrument to assess mental competence, and take up to 4 weeks to reassess mental competence in case of initial non-competence.

The key importance of this study is that it will provide insight in the (cost-)effectiveness of IHT for patients in a psychiatric crisis. The outcome of this study will provide additional evidence to guide the process of further reducing the amount and duration of psychiatric hospitalisations if possible, without jeopardising the quality and safety of care for patients in or on the verge of a psychiatric crisis.

## Abbreviations

(F)ACT: (Functional) Assertive Community Treatment
AUDIT: Alcohol Use Disorder Identification Test (AUDIT)
BES: Relatives evaluation scale (Betrokkenen Evaluatie Schaal (Dutch))
BPRS: Brief Psychiatric Rating Scale
BSI: Brief Symptom Inventory
CAU: Care As Usual
CBS: Statistics Netherlands (Centraal Bureau voor de Statistiek)
CEAC: Cost-Effectiveness Acceptability Curve
CHEERS: Consolidated Health Economic Evaluation Reporting Standards
CIS: Contact, Information and Support
CRT: Crisis Resolution Team
CSQ: Client Satisfaction Questionnaire
DSM: Diagnostic and Statistical Manual of Mental Disease
EHR: Electronic Health Record
EQ-5D-5L: European Quality of Life, 5 Dimensions, 5 levels
GLMM: Generalized Linear Mixed Models
HAMD: Hamilton Depression scale
HoNOS: Health of the Nation Outcome Scale
IC: Informed Consent
ICER: Incremental Cost-Effectiveness Ratio
IHT: Intensive Home Treatment
iMTA: Institute for Medical Technology Assessment
iVICQ: iMTA Valuation of Informal Care Questionnaire
MacCAT-CR: MacArthur Competence Assessment Tool Clinical Research
MANSA: Manchester Short Assessment of Quality of Live
MATE: Measurements in the Addictions for Triage and Evaluation
METc VUmc: Medical Ethics committee of the VU University medical centre
MHCS: Mental Health Confidence Scale
NZa: Dutch Health Care Authority (Nederlandse Zorgauthoriteit (Dutch))
PANNS: Positive and Negative Syndrome Scale
PB: Admission agency (Plaatsing Bureau (Dutch))
PES: Psychiatric Emergency Service
PRODISQ: Productivity and Disease Questionnaire
QALY: Quality Adjusted Life Year
RCT: Randomized Controlled Trial
S&P: Staging and Profiling
SDAS: Social Dysfunction and Aggression Scale
SF-12: Short Form health survey, 12 items
SMI: Severe Mental Illness
SPSS: Statistical Package for the Social Science
TiC-P: Trimbos Institute and Institute of Medical Technology Questionnaire for Costs Associated with psychiatric Illness
TPQ: Treatment Perception Questionnaire
WTP: Willingness To Pay
ZUF: Satisfaction (Zufriedenheit (German))
